# The Relationship Between Concussion and Combat History and Mental Health and Suicide Ideation Among United States Military Veterans—A Pilot Study

**DOI:** 10.3390/brainsci15030234

**Published:** 2025-02-23

**Authors:** Scott L. Bruce, Michael R. Cooper, Carly Farmer, Audrey Folsom, Melanie Fulton, Jana Haskins, Cheryl Knight, Carlitta M. Moore, Amy Shollenbarger, Rashele Wade, Stacy Walz, Rachel Wilkins, Rebbecca Wellborn, Eric West, Kendall Youngman

**Affiliations:** 1 Masters of Athletic Training Program, College of Nursing and Health Professions, Arkansas State University, Jonesboro, AR 72401, USA; 2 School of Nursing, Arkansas State University, Jonesboro, AR 72401, USA; mcooper@astate.edu; 3 Department of Clinical Laboratory Sciences, Arkansas State University, Jonesboro, AR 72401, USA; cafarmer@astate.edu (C.F.); askaggs@astate.edu (A.F.); swalz@astate.edu (S.W.); 4 Department of Social Work, Arkansas State University, Jonesboro, AR 72401, USA; mfulton@astate.edu (M.F.); jhaskins@astate.edu (J.H.); cknight@astate.edu (C.K.); rwade@astate.edu (R.W.); rwellborn@astate.edu (R.W.); 5 Beck Center for Veterans, Arkansas State University, Jonesboro, AR 72401, USA; 6 Access and Institutional Engagement, Arkansas State University, Jonesboro, AR 72401, USA; camoore@astate.edu; 7 Department of Communication Disorders, Arkansas State University, Jonesboro, AR 72401, USA; ashollenbarger@astate.edu; 8 Department of Physical Therapy, Arkansas State University, Jonesboro, AR 72401, USA; rwilkins@astate.edu; 9 Department of Health Sciences & Risk Management–Nutritional Sciences, Arkansas State University, Jonesboro, AR 72401, USA; ewest@astate.edu; 10 Department of Medical Imaging and Radiation Sciences, Arkansas State University, Jonesboro, AR 72401, USA; kyoungman@astate.edu

**Keywords:** suicidality, depression, anxiety, blast exposure, critical prior interval, intrinsic credibility

## Abstract

**Background/Objectives:** Suicides among U.S. military veterans are alarmingly high, driven by factors such as mental health issues, combat exposure, and history of mild traumatic brain injury (mTBI)/concussion. This study aims to examine the relationship between concussion history, combat experience, and their effects on mental health issues and suicide ideation among military veterans. Additionally, this study investigates the impact of post-traumatic stress disorder (PTSD) on these variables. **Methods:** A total of 78 veterans (62 males, 16 females) participated in this study. Participants completed a demographic survey and the Global Well-being Index (GWI) to assess concussion history and residual symptoms. A licensed social worker interviewed the veterans using the 9-Item Patient Health Questionnaire (PHQ-9) for depression, the Generalized Anxiety Disorder survey (7 Items) (GAD-7) for anxiety, and the Columbia-Suicide Severity Rating Scale (C-SSRS) for suicide ideation. A 2 × 2 cross-tabulation analysis examined the relationships between concussion history, combat experience, and outcomes of anxiety, depression, and suicide ideation. PTSD was also assessed as both a predictor and an outcome. Statistical analyses yielded odds ratios (OR) with 95% confidence intervals (CI), Chi-square, and Cramer’s V (V) correlations along with associated *p*-values. **Results:** The combination of concussion history and combat experience strongly predicted either anxiety, depression, or suicide ideation (OR = 7.97, 95% CI: 1.70, 37.44; V = 0.334, (*p* = 0.003)), more than either factor alone. Combat experience was the strongest predictor of PTSD (OR = 11.12, 95% CI: 3.30, 37.47; V = 0.485, *p* ≤ 0.001), both individually and when combined with concussion history. PTSD strongly influenced mental health issues and suicide ideation (OR = 8.16, 95% CI: 1.74, 38.25; V = 0.339, *p* = 0.003). Stratification by PTSD status (positive or negative) affected the relationships between independent and dependent variables. Small cell counts resulted in a wide 95% CI for some ORs, though some statistically significant Fisher’s Exact Test results were observed. Credibility analysis using the critical prior interval (CPI) metric confirmed the intrinsic credibility of the results. **Conclusions:** This study provides insights into the relationships between concussion history, combat experience, and their impacts on mental health issues and suicide ideation among military veterans.

## 1. Introduction

The United States Department of Veterans Affairs (VA) Office of Mental Health and Suicide Prevention reported that between 2001 and 2021, an average of 6374 veterans committed suicide each year, equating to 17.5 veteran suicides per day [1]. These figures could be higher as only 47% of all veterans are enrolled in the VA Healthcare System. The suicide rate among all veterans is 1.5 times greater than that of the general population [2,3]. Among female veterans, the suicide rate is 2.1 times higher than the general population [2].

In contrast, suicide rates among veterans in several Nordic countries are considerably lower. Among 83,584 veterans deployed between 1990 and 2010 in Denmark, Norway, Finland, and Sweden, only 203 of the 1152 deaths (17.6%) were attributed to suicide [4]. In the United Kingdom, among 458,058 veterans who served between 1996 and 2018, only 1086 veterans, or 0.2% of all deaths, were attributed to suicide [5]. Comparing suicide rates in foreign countries, Australia’s veteran suicide rate is 11 suicides per 100,000 veterans [6], Canada’s is 25 suicides per 100,000 veterans [7], and the United Kingdom’s is 8 suicides per 100,000 veterans [8]. The United States’ veteran suicide rate has ranged from 32.6 to 33.9 suicides per 100,000 veterans in the 2020s [1,9].

Several causes of suicidality have been cited in the literature. Schafer et al. performed a comprehensive meta-analysis and identified over 50 independent variables studied across various research [3]. Some of these risk factors include post-traumatic stress disorder (PTSD) [3,10,11]; mental health issues such as depression [3,12], anxiety, mania, bipolar disorder, psychosis, anger or aggression; substance/alcohol use disorders [3]; social, environmental issues [3,13]; combat exposure [3]; adverse childhood events [13,14]; prior self-injurious thoughts and behaviors; financial and legal problems; witnessing the death of the enemy; death of fellow service personnel or grave injury to oneself or a peer; access to lethal means [3]; and mild traumatic brain injury (mTBI) or concussion [3,11]. Of importance, suicidality is rarely caused by a single factor; often, multiple causes are interrelated or concomitant [15].

Mental health issues are prevalent among military personnel. A study of 2449 veterans’ primary health records found that 38% (n = 938) had at least one common mental disorder coded in their records. The most common mental health issues recorded were depression (17.8%, n = 437), anxiety (15.0%, n = 367), and PTSD (3.4%, n = 83). Females had a higher prevalence of depression (25.8% vs. 16.7%) and anxiety (25.5% vs. 13.5%) compared to their male counterparts, while males had a higher prevalence of PTSD (3.7% vs. 1.3%) due to the volume of combat-related traumas experienced [16]. Another study found the lifetime prevalence of PTSD in the United States to be 6.1%, with a higher prevalence among genders: 11.5% for men and 18.9% for women [17].

The Department of Defense reported over 468,000 TBIs from 2000 to November 2022, with 82.3% being mild TBIs (mTBI) [11,18]. The current literature suggests no difference between military blast-related TBIs and non-blast-related TBIs [11,19]. The American Congress of Rehabilitation Medicine (ACRM) defines mTBI as an injury to the brain resulting from a “biomechanically plausible mechanism of injury” with specific clinical signs and symptoms, neuroimaging evidence, and no confounding factors that fully account for the clinical findings [20,21].

The demographics of college and professional athletes are like those of service members in terms of age, height, weight, physical abilities, and energy expenditure. Therefore, the Concussion in Sport Group (CISG) definition of a sport-related concussion can be applied to both athletic and military populations. A sport-related concussion is defined as a TBI caused by a direct blow resulting in an impulsive force transmitted to the brain, initiating a neurotransmitter and metabolic cascade that may present symptoms immediately or evolve over time [20,22]. Both the ACRM and CISG agree that an individual meeting the ACRM definition for mTBI has suffered a concussion [20,21]. However, unlike earlier sports medicine practices, the ACRM applies evidence-based criteria to qualify a TBI as “mild”. For a TBI to be considered mild, there cannot be indicators such as loss of consciousness greater than 30 min, a Glasgow Coma Scale score below 13 post-injury, or post-traumatic amnesia lasting over 24 h [20,21].

Studies have found that individuals who sustain a sport-related concussion are at greater risk of depression, anxiety, and suicidality [23,24,25,26,27]. The same is true for military veterans, with PTSD often adding to the mental health burden [10,15,28,29]. Brenner and colleagues found a significant increase in depression (68%), anxiety (75%), and PTSD (96%) after suffering a concussion [15]. Fralick et al. conducted a systematic review and meta-analysis, finding a relative risk of suicide after a concussion or mTBI ranging from 1.73 to 3.02, with an overall relative risk of 2.03 (95% CI 1.47, 2.80) [28]. For military veteran studies, relative risks ranged from 1.10 to 1.98, though some studies were outside the five-year window for this paper [30,31].

Fisher et al. reported the prevalence of suicide ideation and behavior among veterans diagnosed with mTBI and PTSD, with lifetime suicide ideation at 40%, current suicide ideation at 25%, and lifetime suicide behavior at 19%. For veterans with only PTSD, the prevalence was 29%, 11%, and 11%, respectively. For those with only mTBI, the prevalence was 14%, 1%, and 1%, respectively [29]. A 2019 study by Hostetter et al. examined VA records of over 1.1 million veterans, finding a hazard ratio of 2.19 (95% CI: 2.02, 2.37) for suicide among those with mTBI compared to those without [32]. Veterans with mTBI had higher prevalence rates of depression (68.1% vs. 60.8%), anxiety (47.6% vs. 38.7%), and PTSD (62.8% vs. 39.1%) compared to those without a history of mTBI [32].

Veterans may be exposed to blasts from various sources, including combat, training, or occupational incidents [33,34]. Although the exact mechanics of blast-induced head trauma are not fully understood, a commonly accepted sequela is that brain injuries result from such trauma [11,35]. Heath et al. described the clinical presentation of blast-induced TBI, noting that veterans often experience multiple concussions over their careers, leading to a cumulative effect on mental health [11].

These findings emphasize the necessity to address the mental health needs of veterans with a history of mTBI and to develop effective interventions that reduce the risk of suicidality. This requires a comprehensive approach to include early identification, monitoring, and treatment of mental health issues, as well as providing support and resources to help veterans cope with their experiences and challenges.

### Purposes and Hypotheses

The purpose of this study was to examine the relationship between concussion history, combat experience, and the effect on mental health and suicide ideation among military veterans. A second purpose was to investigate the relationship concussion history and combat experience had on PTSD, and the relationship mental health issues and suicide ideation had on PTSD among military veterans.

This study had three hypotheses:

**H1.**  Multiple concussions would be a strong predictor of mental health issues and suicide ideation among military veterans.

**H2.**  The addition of combat experience with either one of the head injury histories would be a strong predictor of mental health issues and suicide ideation among military veterans.

**H3.**  Adding PTSD to the analyses would be an added strong predictor of mental health issues and suicide ideation among military veterans.

## 2. Materials and Methods

### 2.1. Study Design

This cross-sectional pilot study was reviewed and approved by the university’s Institutional Review Board. Data for this study were gathered as part of the Veterans’ Suicide Prevention Project (VSPP), an interprofessional group of allied health professionals and researchers. The VSPP was charged with studying the mental health and suicide ideation issues of military veterans in northeast Arkansas, with the eventual goal of taking the lessons learned and expanding the project to the state of Arkansas, the Delta region of the country, and perhaps even nationally.

### 2.2. Participants

Participants were recruited from various military veteran organizations in northeast Arkansas and through the veterans’ center on our campus. Veterans were scheduled for their data collection in groups of four veterans every 20 min. Data were collected over two different days. Veterans were scheduled to report to the testing location. Each veteran completed data collection in approximately two hours. Any person 18 years of age and older who identified as a veteran of the U.S. military qualified for participation in the study. Exclusion criteria were anyone not a U.S. veteran. No requirements or quotas were imposed on the demographics for this study. No qualifiers were imposed upon the participants other than their veteran status.

### 2.3. Survey Instruments

Participants provided informed consent before taking part in this study. They completed two surveys electronically, either on an iPad or their personal cell phone, accessed via QR code. A demographic survey collected information about their personal and military history. This survey included a “Yes/No” question about combat history, disability status, and type of disability (post-traumatic stress disorder (PTSD) was a common response).

The Global Well-being Index (GWI) was used to assess residual concussion-related symptoms, concussion history, and physical limitations they may be currently experiencing. This 5-item instrument has a maximum score of 50 points, with a higher score indicating a greater symptom burden. Concussion history was determined by a “Yes/No” question: “Have you ever experienced a blow to the head that caused altered mental status, including any of the following: feeling confused, feeling dazed, feeling stunned, seeing stars, or felt like you got your bell rung”? A “Yes” response activated a follow-up question about the number and timing of most recent concussions.

Each participant was interviewed by a licensed social worker to assess depression, anxiety, and suicide ideation. Depression was measured using the 9-Item Patient Health Questionnaire (PHQ-9), which asks participants to rate the frequency of nine symptoms over the last two weeks—scored 0 = “Not at all”, 1 = “Several days”, 2 = “More than half the days”, and 3 = “Nearly every day”. The PHQ-9 has demonstrated excellent internal reliability (Cronbach’s alpha = 0.86, 0.89) and good test–retest reliability (Intraclass Correlation Coefficient (ICC) = 0.84), with established construct and criterion validity [36].

Anxiety was assessed using the Generalized Anxiety Disorder (7 Items) (GAD-7) survey, which asks participants to rate the frequency of seven symptoms over the last two weeks and is scored from 0 (not at all) to 3 (nearly every day). The GAD-7 has shown good internal reliability (Cronbach’s alpha = 0.92) and test–retest reliability (ICC = 0.83), with established construct and criterion validity [37].

Suicide ideation was assessed using the Columbia-Suicide Severity Rating Scale (C-SSRS), a six-item survey instrument asking participants about suicidality during the past month and at their worst point in life [38]. The C-SSRS is considered the “gold-standard” for assessing lifetime suicidal ideation and behavior [29]. Responses are “Yes” or “No” and are color-coded to help clinicians identify at-risk individuals. Cronbach’s alpha for the C-SSRS has ranged from 0.73 to 0.94, with established convergent and divergent validity [38].

### 2.4. Statistical Analyses

The dependent variables in this study were depression, anxiety, and suicide ideation. Depression was classified based on a PHQ-9 score of ≥10 points, indicating “Moderate” to “Severe” depression [36]. Anxiety was classified based on a GAD-7 score of ≥10 points, indicating “Moderate” to “Severe” anxiety [37]. Suicide ideation was indicated by a “Yes” response to either item two [39] or item six on the C-SSRS [40]. Participants were dichotomized as positive or negative for these variables, with positive cases coded as “1” and negative cases coded as “0”. Univariable and multivariable analyses were conducted for all variables. For the multivariable analysis, when grouped together, depression, anxiety, and suicide ideation were referred to as the “Big 3”.

Independent variables of interest included concussion history, multiple concussion history, and combat experience. Participants were considered “positive” for a concussion history if they responded positively to the concussion question on the GWI. A multiple concussion history was indicated if they reported experiencing two or more concussions. Combat experience was indicated by acknowledging service in a combat area. Positive classifications were coded “1”, and all others were coded “0”. Univariable and multivariable analyses were conducted for these predictor variables.

The relationship between a PTSD diagnosis and other variables was also examined. Veterans with a PTSD diagnosis were coded as “1”, and those without PTSD as “0”. Initial analyses compared PTSD diagnosis to both dependent and independent variables individually and in combination.

Cross-tabulation tables (2 × 2) were generated for all variable combinations. Metrics of interest included odds ratios (OR) with 95% confidence intervals (95% CI), Chi-square (*χ*^2^) test for independence, Fisher’s Exact Test (1-sided), and Cramer’s V (V) test for strength of the relationship between variables alongside the advocacy or skepticism limit. Operationally, we considered ORs ≥ 2.0 and V of ≥ 0.20 as clinically significant. Qualitative descriptors for V coefficients are listed in Table 1. The alpha level for all statistical tests was set at ≤0.05. In cases where a cell in the 2 × 2 cross-tabulation table yielded zero (0) cases, 0.5 was added to each cell, following the method recommended by Hosmer, Lemeshow, and Sturdivant (2000) [41]. All analyses were conducted using IBM SPSS Statistics version 29.0.1.0 (Armonk, NY, USA).

The American Statistical Association has expressed concern about the heavy reliance on *p*-values, advocating for greater use of confidence intervals, Bayesian methods, and false discovery rates [42]. Matthews (2018) advocates for “Analysis of Credibility” using metrics to generate a “Critical Prior Interval” (CPI) to place results in the context of existing knowledge [43]. This study used the CPI approach to compare ORs to the “advocacy limit” or “skepticism limit”, aiding in the representation of uncertainty and the exclusion of a null effect [44,45,46].

The advocacy limit (AL) was applied when the OR was not statistically significant, while the skepticism limit (SL) was used when the OR was statistically significant. For OR > 1.0, the lower boundary was fixed at 1.0, with the upper limit as the AL or SL outcome. For OR < 1.0, the upper boundary was fixed at 1.0, with the lower limit as the AL or SL outcome. This ensured that the CPI encompassed 95% of the prior distribution, making the findings credible evidence of a positive effect. Weak evidence produced broad intervals, while stronger evidence produced narrower intervals. When the lower limit of the SL 95% CI < 1.0, calculating the SI was not possible [44].

Univariable analyses included examining the outcome variables with each of the predictor variables. Multivariable analyses included 82 different variable combinations. PTSD was evaluated as an outcome variable and as a predictor variable. Table 2 provides a list of the analyzed variables and combinations.

**Table 1:** Qualitative descriptors for the strength of the association with the corresponding effect size [47].

Cramer’s V Coefficients	Category
>0.25	Very Strong
>0.15	Strong
>0.10	Moderate
>0.05	Weak
>0	None or Very Weak

**Table 2:** Variable combinations used in the analysis.

Outcome Variable Combinations	Predictor Variable Combinations
PHQ-9 ^a^	Combat ^e^
GAD-7 ^b^	Concussion Hx ^f^
Suicide Ideation ^c^	Multi-Concussion Hx ^g^
Any 1 of the Big 3 ^d^	Both Concussion Hx and Combat Exp
Any Combo of 2 of the Big 3	Either Concussion Hx or Combat Exp
All 3 of the Big 3	Both Multiple Concussion Hx and Combat
Both PHQ-9 and GAD-7	Either Multiple Concussion Hx or Combat
Either PHQ-9 or GAD-7	
Both PHQ-9 and Suicide Ideation	PTSD ^h^ was assessed as both a predictor and an outcome variable
Either PHQ-9 or GAD-7	
Both GAD-7 and Suicide Ideation	
Either GAD-7 or Suicide Ideation	

^a^ Positive for depression as per a 9-Item Patient Health Questionnaire (depression) ≥10. ^b^ Positive for anxiety as per a Generalized Anxiety Disorder (7 Items) score of ≥10. ^c^ Positive for suicide ideation as per the Columbia-Suicide Severity Rating Scale—“Yes” response to item 2 or item 6 of the survey. ^d^ Big 3—combination of depression, anxiety, and suicide ideation. ^e^ Combat experience. ^f^ Concussion history. ^g^ Multiple concussion history. ^h^ Post-traumatic stress disorder.

### 2.5. Use of Artificial Intelligence

Generative artificial intelligence (AI) was used to edit this manuscript. The original text was authored by the researchers and then edited using ChatGPT (OpenAI, San Francisco, CA, USA, GPT-4-turbo variant, 2023). ChatGPT was prompted with “Edit this text”. Due to the size of this manuscript, ChatGPT had to be asked to edit the manuscript in sections. The same prompt was used each time. The revised version was checked against the original to ensure accuracy of citations and content integrity. The AI-assisted revision took place on 28 December 2024. The authors are responsible for the integrity of the content provided.

## 3. Results

One hundred veterans volunteered and were scheduled to participate in data collection. A total of 78 United States military veterans (62 males, 16 females) participated in this study; 22 veterans did not report for data collection. Demographic data are presented in Table 3. Participants represented all branches of the military. No requirements or quotas were imposed on the demographics for this study. Military-related demographics are provided in Table 4.

During our analysis, we encountered only one combination where a cell had zero cases reported in one of the 2 × 2 cells (“Any 1 of the Big 3 × Both Multiple Concussion Hx and Combat Exp”). The extrapolated results showed this combination had the highest OR but also the widest 95% CI (OR = 13.04, [95% CI: 0.740, 229.90]; V = 0.217, [*p =* 0.17]). Caution is advised when interpreting this outcome due to its extrapolated nature. Table 5 presents variable combinations that had statistically significant *p*-values on the Fisher’s Exact Test (one-sided) (*p ≤* 0.05).

### 3.1. Impact of Concussion History and Combat Experience

Veterans with both a history of concussion and experienced combat were at a much higher risk for mental health issues, including suicide ideation. ORs ranged from 7.97 to 2.79, with Cramer’s V correlations ≥ 0.217 (Table 5). Combat experience was a consistent predictor, appearing in 94.1% of statistically significant groupings. Concussion history appeared in 41.1% of the groups. A history of multiple concussions was a predictor in 36.3% of the groups. When assessed individually, combat and concussion history were the only two predictors that produced statistically significant results, and they were only significant for 5 of the 17 groups (29.4%). Combat experience was significant for four of those five combinations, with ORs ranging from 4.40 to 3.25 and very strong Cramer’s V correlations all >0.250.

The strongest variable combination that was not extrapolated was “Any 1 of the Big 3 × Both Concussion Hx and Combat Exp”, with an OR = 7.97 (95% CI: 1.70, 37.44) and a V = 0.334. This suggests that veterans with both a history of concussion and combat experience had eight times greater odds of experiencing depression, anxiety, or suicidal ideation compared to veterans without this combination of risk factors. The relationship between these variables and the Big 3 was strong.

Suicide ideation was the only outcome variable to be statistically significant as an individual dependent variable. In these cases, combat was a predictor in three ways: combat alone, either a history of multiple concussions or combat, and both concussion history and combat. The ORs ranged from 3.40 to 3.18, with Cramer’s V correlations ranging from 0.274 to 0.264.

For combinations with Fisher’s Exact Test (one-sided) (*p* ≤ 0.05), the SL was calculated to determine intrinsic credibility. Ten variables with calculable SL values fell within the 95% CI of the ORs, confirming positive effects for *p*-values ≤ 0.05 if prior evidence supports effect sizes in the skepticism CPI of 1.0 and the SL value (1, SL). The strongest combination, “Any 1 of the Big 3 × Both Concussion Hx and Combat Exp”, showed strong evidence for the effect despite low cell counts, with tight CPIs making the skeptical challenge more demanding [44].

### 3.2. Influence of Post-Traumatic Stress Disorder

PTSD’s link to mental health issues and suicide risk, similar to what the literature states, was strong in our sample [16,29,48]. Twenty-five veterans acknowledged PTSD (25/78 = 32.1%). PTSD can either be an outcome or it can be a predictor in our study. We examined the impact of PTSD as an outcome and a predictor. In both univariable and multivariable analyses, 19 PTSD-related combinations were examined. Of these, 17 were statistically significant, with strong ORs and lower 95% CI margins > 1.0. PTSD × concussion history and PTSD × multiple concussion history did not meet these criteria (Table 6).

The connection between PTSD and combat experience is unmistakably connected (OR = 11.12, 95% CI: 3.30, 37.47; V = 0.485, *p* < 0.001). This indicates that veterans with combat experience had over 11 times greater odds of developing PTSD than those without combat experience, which is consistent with the literature [49,50]. The Cramer’s V was very strong (0.485). As with the analyses of the variable combinations in Table 6, combat is a statistically significant predictor of PTSD.

PTSD is a strong predictor of mental health issues, specifically depression, anxiety, and suicide ideation. The combination of PTSD × either depression or suicide ideation and PTSD × either anxiety or suicide ideation (OR = 8.16, 95% CI: 1.74, 38.25; V = 0.339, *p* = 0.003) shows that veterans with PTSD had over eight times greater odds of experiencing depression or suicidal ideation and anxiety or suicidal ideation compared to those without PTSD.

The next strongest combination was “Any 1 of the Big 3” (OR = 6.43, 95% CI: 1.36, 30.28; V = 0.293, *p* = 0.010). The relationship between PTSD and suicidal ideation was also strong (OR = 5.64, 95% CI: 1.84, 17.31; V = 0.360, *p =* 0.001), with PTSD increasing the odds of suicidal ideation by over five and a half times. Examining PTSD as a predictor of mental health issues and suicide ideation found the relationship to also be very strong. This was true regardless of univariable or multivariable analyses. Cramer’s V correlations, except for two, were very strong, ranging from 0.238 to 0.485 (Table 1). The SL was calculated for the top 17 combinations, indicating strong evidence for tighter CPI. Two variables with *p*-values > 0.05 had AL calculated instead. The PTSD × Multi-Concussion Hx combination had a narrow AL, but the PTSD × Concussion Hx combination had a wide AL, suggesting limited added value to prior evidence [44].

Stratifying data based on PTSD diagnosis produced three outputs: PTSD positive, PTSD negative, and PTSD total for 165 total combinations. We focused on PTSD-positive cases with an OR ≥ 1.5, as shown in Table 7. Stratification led to smaller cases in the 2 × 2 cross-tabulation table cells and led to identical 2 × 2 cell counts for several combinations, yielding identical outputs. The strongest ORs for PTSD Positive cases were “Any 1 of the Big 3 × Multi Concussion Hx” and “Either Anxiety or Suicide Ideation × Multi Concussion Hx” (OR = 4.60, 95% CI: 0.199, 106.30; V = 0.261, *p* = 0.191). Only two Fisher’s Exact Test (one-sided) *p*-values were statistically significant, with SL calculated for one variable. “Any 1 of the Big 3 × Both Concussion Hx and Combat Exp” for PTSD-negative cases had an AL value of 6,226,740, suggesting weaker evidence for a positive effect given the wide AL and lower OR boundary close to 1.0. For “Either Anxiety or Suicide Ideation × Both Concussion Hx and Combat Exp”, SL could not be calculated due to the lower boundary of the OR’s 95% CI being < 1.0.

The AL for “Either Anxiety or Suicide Ideation × Multi Concussion Hx” for PTSD-negative cases was 0.473, with an OR of 0.833, requiring the AL to be inverted from “1, AL” for OR > 1 to “AL, 1” for OR < 1. Stratification was responsible for the smaller than usual numbers of cases in the 2 × 2 cross-tabulation table cells, causing all of the ORs, except for one, to have a lower limit of the 95% CI to be < 1.0. The one exception was PTSD negative for “Any 1 of the Big 3 × Both Concussion Hx and Combpat Exp”, but its OR was only 1.02. Another result of the smaller number of cases is the AL metrics to vary greatly.

## 4. Discussion

Our study aimed to examine the relationship between concussion history, combat experience, and their effects on mental health issues and suicide ideation among military veterans. Additionally, we investigated the impact of PTSD on these variables, both as an independent variable and as a dependent variable. Suicidality has numerous and highly specific causes for each individual [3]. Mental health issues constitute a significant proportion of the reasons for contemplating suicide [3,12,13,14].

### 4.1. Hypotheses

Multiple concussions as predictors of mental health issues and suicide ideation. Of the 17 combinations reported in Table 5, only six included multiple concussion history. While this is one-third of the combinations, four of these had low cell counts, with fewer than five cases in one cell of the 2 × 2 cross-tabulation table. The strongest predictor was based on an extrapolated result due to a zero-case cell. As a single variable, multiple concussion history was not a robust predictor, but when combined with combat experience, multiple concussion history became a more effective predictor.

Combat experience combined with head injury histories. Combat experience, whether as a single variable or in combination with head injury variables, was a strong predictor of mental health issues and suicide ideation among military veterans. Combat experience alone was a good predictor of mental health issues (OR = 3.27) and suicide ideation (OR = 3.25).

PTSD as a predictor of mental health issues and suicide ideation. PTSD was a strong variable, whether as a dependent or independent variable. PTSD is often an outcome of combat, and its relationship with head injury history strengthens when combined with combat experience. The smallest odds ratio reported for PTSD was 3.05 (Table 6).

### 4.2. Concussion, Mental Health, and Suicidality

Combat experience and explosive blast exposure are linked to PTSD and concussion [51]. Repeated low-level blast exposure appears to cause mTBI/concussion effects, even if high-level blasts cause more immediate TBI. These low-level exposures can result in persistent concussion symptoms and potentially lead to mental health issues or sensory losses (e.g., hearing, sight, touch), which can be particularly frustrating for veterans [51,52]. The similarity in symptoms between blast-related head injuries and PTSD, such as dizziness, visual symptoms, cognitive dysfunction, sleep disturbances, depression, anxiety, fatigue, and irritability, further complicates diagnosis and treatment.

Regardless of the cause of the brain trauma, the sequala is similar to the initiation of a metabolic cascade in which the chemicals of the brain become mixed together, like a shaken bottle of Italian salad dressing. The trauma causes cell damage and the leakage of proteins into the blood and cerebrospinal fluid. The mTBI results in a decrease in cranial blood flow and in available glucose and a dramatic increase in elements essential for neural activity (i.e., potassium, calcium, glutamate, and sodium) [53]. Much like that bottle of Italian salad dressing that takes approximately 7–10 days for ingredients in the Italian dressing to separate themselves, it takes 7–10 days or longer for the chemicals in the brain to be sorted and equilibrium of the elements and neurotransmitters to return to “normal” levels. Neuroinflammation is a consequence of the trauma and mixing of chemicals in the brain [53,54]. Several studies have identified several biomarkers indicating brain trauma, such as microglia, glial fibrillary acidic protein (GFAP), neurofilament light chain, amyloid beta 40 and 42, and microRNA [55,56]. The number of biomarkers, the concentration of biomarkers, the timing of their appearance, and the vast variety of everybody’s brains provide a rich research platform from which numerous papers have been published and continue to be published, but such a discussion is outside the scope of this project.

The mechanism of injury causing the concussion or the mTBI does not matter since the clinical presentation is similar regardless of the setting. In the military population, service personnel obviously often encounter serious traumas not seen in other areas of society. Military veterans have a higher prevalence of mental health issues. Depression, anxiety, PTSD, and substance abuse are the most commonly reported conditions [16]. These are typical mental health issues that proceed with suicide ideation [28]. Those individuals suffering a concussion/mTBI tend to also suffer from these mental health conditions. Hostetter et al. report that 60% of veterans diagnosed with PTSD had a history of concussion compared to only 15% of those without a concussion history [32]. Those veterans with a concussion history also had a higher prevalence of depression, anxiety, and other psychotic disorders [28,32,48]. Hence, those with a history of concussion are at a higher risk for suicidality, as much as twice as likely for suicide [28,32]. The results of our study showed much the same thing; although we did not see the suicidality larger studies did, the connection between a history of concussion and mental health issues was present.

### 4.3. Chronic Traumatic Encephalopathy

Consideration for the presence of chronic traumatic encephalopathy (CTE) in the veterans of our sample is a possibility. A thorough discussion of CTE is outside the scope of this article. However, CTE is a complicated issue with no consensus regarding the presence of CTE in military veterans. 

CTE is a progressive deterioration of brain function resulting in behavioral alterations, dementia, depression, gait abnormalities, personality changes, and speech abnormalities, which has been associated with “atrophy of multiple cortical brain regions.” [57]. There is a belief that repetitive head impacts are responsible for the development of CTE, but because CTE can only be diagnosed post-mortem, no definitive cause-and-effect conclusion has been determined [57,58,59]. The literature does show a relationship between contact, collision, or contact sports athletes, military personnel, and victims of violent crimes [57,58,59,60,61].

Military personnel are exposed to repetitive explosions, elevating their risk for repetitive head impacts. The pressure wave produced by a blast causes acceleration and/or rotation of the brain, similar to head impacts in some sports [34,58]. Traumatic brain injuries resulting from explosions are part of the “neuropsychiatric spectrum disorder that clinically overlaps with chronic traumatic encephalopathy.” [58]. Priemer and his colleagues examined 225 brains of military veterans for CTE. They reviewed the medical records of these veterans to determine past medical history of mTBI, contact sport exposures, and other mental health disorders. They found CTE in only 10 of the 225 brains examined (4.4%). Only three had a history of blast exposure. Three of the brains with CTE had suffered a concussion from some other military impact mechanism. Of the 225 brains examined, 60 of those individuals were contact sports participants; all 10 CTE cases had participated in contact sports. Priemer et al. concluded that CTE appeared to be more related to contact sport exposure rather than military blast exposure but cautioned the reader that the small sample size and large CIs for the risk ratios “preclude causal conclusions” [61].

### 4.4. Study Implications

Our study was a descriptive pilot project involving 78 military veterans. While many of our outcomes were statistically significant from a frequentist perspective, this does not necessarily mean they are true. Conversely, from a Bayesian perspective, several odds ratios were robust and statistically significant using the Fisher Exact Test (one-sided), providing evidence that our results are true [44]. Some may see the non-significant results or the lower boundary of the 95% CIs below 1.0 and conclude our results are false. Neither perspective is entirely correct nor incorrect.

This pilot study is the initial examination of the issues facing military veterans. Future efforts will focus on prevention and treatment or intervention studies to attempt to reduce the high number of daily suicides among military veterans. For our findings to be truly meaningful to a broader population, a much larger study involving not only a greater number of veterans but a more diverse veteran population is needed. Expanding from northeast Arkansas, and the delta region will be needed if we are to affect a broader number of veterans.

### 4.5. Analysis of Credibility

The analysis of credibility proposed by Matthews [44] indicates that our research is on the right track. We found several statistically significant outcomes. For statistically significant results, the skeptical limit provides credible evidence to support further research. For non-significant results, the critical prior interval shows credible evidence of a “non-zero effect size” despite *p*-values > 0.05. In this study, we relied on ORs as our output metric, which is what the skeptical and advocacy limits are derived from. An examination of these limits finds several promising results for further study. Using this approach, we can see that our data and results are intrinsically credible.

### 4.6. Study Limitations

There were limitations to our study. We did not directly ask for a PTSD diagnosis, instead inferring PTSD from other data on disability status. We also did not collect information on medication histories, which could have influenced mental health outcomes. We did not ask the participants about their mental health histories except for what was provided by the PHQ-9 and GAD-7. We did not directly ask about musculoskeletal injuries outside of the veterans’ military service; we were able to determine physical limitations through the GWI survey. There were 16 female veterans in this study, and because this was a pilot study and there were only 78 total participants, we did not stratify the data based on sex. It is possible the findings could have been skewed as a result. The small data set left us with some large 95% CIs and unstable results. The data presented here are not a comprehensive summary of all data gathered from the 78 veterans, and other collected data could potentially impact our reported results.

### 4.7. Clinical Implications

Our findings provide valuable information for clinicians working with veterans about their concussion history, mental health issues, and suicide ideation. While concussion in athletes has been extensively studied, the long-term effects of brain trauma in veterans are not yet fully understood. Although PTSD in athletes can occur, their stress disorder does not rise to the level of trauma experienced by combat veterans. Further investigation into PTSD is crucial to understanding mental health issues and suicide ideation in military veterans.

### 4.8. Generalizability

Caution should be exercised when examining the results of this study. Although the research team was pleased to get 78 veterans to participate, the sample was made up of veterans from northeast Arkansas. Due to the small sample size and regionality of the sample, one would have difficulty drawing valid conclusions about the general population of United States veterans. As a pilot study, it is a good start toward continuing to examine the mental health issues and suicidality of veterans. Further, our study provides a foundation from which future research can be explored.

## 5. Conclusions

Our research underscores the importance of considering both physical and psychological trauma in veterans. The interplay between concussion history, combat experience, and PTSD significantly affects mental health outcomes, necessitating a multifaceted approach to veteran care. A history of multiple concussions was an effective predictor for mental health issues when combined with combat experience. Combat experience was a strong indicator of mental health issues (OR = 3.27) and suicide ideation (OR = 3.25). PTSD was noted as a strong variable and strengthened when combined with combat experience.

There are several limitations in this study, as noted above. Future research planned will utilize the lessons learned from this study. For example, the initial veteran questionnaire utilized is being updated to capture more specific data in areas we failed to inquire about, such as medication history, injuries outside of their military service, and disability conditions.

The clear need for more biopsychosocial research and exploration related to the trauma veterans experience became more apparent during this study. As an example of this, during our data collection, the research team identified one veteran in crisis, plus two additional veterans were identified as being positive for suicide ideation. In all three cases, the veterans were provided the assistance they needed. As stated earlier, 17.5 veterans and likely more commit suicide each day in the United States [1]. The magnitude of our efforts was demonstrated clearly by the needs of these three veterans. This experience further emphasized the need for care within the military veteran community to address the biopsychosocial causes of suicidality in military veterans.

Future research with a larger and more diverse sample is needed. A more thorough assessment of PTSD, an examination of female veterans specifically and their unique challenges, an exploration of the predictability of standardized patient-reported outcome measures for suicidality, and specific intervention studies to determine if the effects of mTBI and mental health issues can be improved are all areas for further investigation.

This study was just the beginning of understanding the reasons behind suicide ideation and mental health issues in military veterans. Much work remains. Concussion takes a multidisciplinary, interprofessional approach to the assessment, treatment, and management of the trauma. It appears the mental health issues faced by military veterans will also require a multifaceted and interprofessional approach to remedy.

## Figures and Tables

**Table 3:** Demographic data by sex.

Mean (±sd)	Males (n = 62)	Female (n = 16)
Age (yrs)	55.40 (±17.03)	46.06 (±15.30)
Height (cm)	179.40 (±7.29)	170.18 (±13.73)
Weight (kgs)	99.14 (±20.64)	80.27 (±15.07)
Body Mass Index (BMI)	30.73 (±5.56)	28.18 (±6.60)

**Table 4:** Summary of military-related demographics by sex.

Breakdown of Branches of the Service	Male	Female	Total
U.S. Air Force	6	3	9
U.S. Army	30	9	39
U.S. Marine Corps	9	1	10
U.S. Navy	11	2	13
National Guard	6	1	7
**Classification upon Separation**			
Officer	11	1	12
Enlisted	51	15	66
**Combat Experience**			
Combat Experience	30	8	38
No Combat Experience	32	8	40
**Number of Years in Service**			
Mean	12.7	10.4	12.2
(±sd)	(±10.5)	(±8.1)	(±10.0)
Minimum	<1 yr	1 yr	
Maximum	42 yrs	26 yrs	

**Table 5:** Results of 2 × 2 cross-tabulation analyses for variable combinations.

Variable	OR ^a^	95% CI ^b^ for OR	χ^2 c^	*p*-Value for χ^2^	Fisher’s Exact Test (1-Sided) *p*-Value	V ^d^	*p*-Value for V	SL ^e^ (1, SL)
Any 1 of the Big 3 ^f^ × Both Multiple Concussion Hx ^g^ and Combat Exp. ^h,^*	13.04	0.740, 229.90	5.75	0.017	0.011	0.217	0.017	N/A
Any 1 of the Big 3 × Both Concussion Hx ^i^ and Combat Exp. **	7.97	1.70, 37.44	8.69	0.003	0.002	0.334	0.003	5.61
Either Anxiety ^j^ or Suicide Ideation ^k^ × Both Multiple Concussion Hx and Combat Exp. **	6.57	0.803, 53.79	3.90	0.048	0.042	0.224	0.048	N/A
Either Depression ^l^ or Anxiety × Both Concussion Hx and Combat Exp. **	6.03	1.61, 22.65	8.25	0.004	0.003	0.325	0.004	4.19
Either Anxiety or Suicide Ideation × Both Concussion Hx and Combat Exp. **	6.03	1.61, 22.65	8.25	0.004	0.003	0.325	0.004	4.19
Either Depression or Anxiety × Both Multiple Concussion Hx and Combat Exp. **	4.71	0.968, 22.97	4.24	0.040	0.036	0.233	0.04	N/A
Any 1 of the Big 3 × Combat Exp.	4.40	1.42, 13.67	7.14	0.008	0.007	0.302	0.008	3.81
Either Anxiety or Suicide Ideation × Combat Exp.	4.36	1.49, 12.74	7.81	0.005	0.005	0.316	0.005	3.14
Any Combo of 2 of the Big 3 × Both Multiple Concussion Hx and Combat Exp. **	3.89	0.979, 15.45	4.11	0.043	0.041	0.229	0.043	N/A
Suicide Ideation × Either Multiple Concussion Hx or Combat Exp.	3.40	1.24, 9.36	5.87	0.015	0.015	0.274	0.015	4.36
Either Depression or Anxiety × Combat Exp.	3.27	1.17, 9.19	5.30	0.021	0.019	0.261	0.021	6.05
Suicide Ideation × Combat Exp.	3.25	1.28, 8.25	6.33	0.012	0.011	0.285	0.012	3.33
Suicide Ideation × Both Concussion Hx and Combat Exp.	3.18	1.18, 8.58	5.43	0.020	0.017	0.264	0.02	5.21
Either Depression or Anxiety × Concussion Hx	3.05	1.13, 8.23	5.00	0.025	0.024	0.253	0.025	7.04
Either Depression or Anxiety × Either Concussion Hx or Combat Exp.	2.79	0.980, 7.96	3.83	0.050	0.048	0.222	0.05	N/A
Either Anxiety or Suicide Ideation × Either Concussion Hx or Combat Exp.	2.79	0.980, 7.96	3.82	0.050	0.048	0.222	0.05	N/A
Any 1 of the Big 3 × Either Multiple Concussion Hx or Combat Exp.	2.79	0.980, 7.96	3.83	0.050	0.048	0.222	0.05	N/A

^a^ Odds ratio; ^b^ Confidence interval; ^c^ Chi-squared; ^d^ Cramer’s V test, ^e^ Skepticism limit (based on the Fisher Exact Test (one-sided) *p*-value); ^f^ Big 3—combination of depression, anxiety, and suicide ideation; ^g^ History; ^h^ Combat experience; ^i^ Concussion history; ^j^ Positive for anxiety as per a Generalized Anxiety Disorder (7 Items) score of ≥10; ^k^ Positive for suicide ideation as per the Columbia-Suicide Severity Rating Scale—“Yes” response to item 2 or item 6 of the survey; ^l^ Positive for depression as per a 9-Item Patient Health Questionnaire ≥ 10. * Variable combination had one cell with zero (0) cases in it, and 0.5 was added to each cell to allow estimated results to be calculated. ** Variable combination had one cell of the 2 × 2 table with <5 cases (N/A)—with the lower limit of the OR 95% CI < 1, it was not possible to calculate the SL for those variables.

**Table 6:** A 2 × 2 cross-tabulation analysis for PTSD by variable combinations.

Variable	OR ^a^	95% CI ^b^ for OR	χ^2 c^	*p*-Value for χ^2^	Fisher’s Exact Test (1-Sided) *p*-Value	V ^d^	*p*-Value for V	SL ^e^ (1, SL)
PTSD ^f^ as an outcome in relation to the predictor variables								
PTSD × Combat Exp ^g^	11.12	3.30, 37.47	18.33	0.000	0.000	0.485	0.000	2.03
PTSD × Either Multiple Concussion Hx ^h^ or Combat Exp	8.16	1.74, 38.25	8.95	0.003	0.002	0.339	0.003	5.37
PTSD × Both Multiple Concussion Hx and Combat Exp	6.89	1.87, 25.44	9.90	0.002	0.003	0.356	0.002	3.31
PTSD × Either Concussion Hx ^i^ or Combat Exp	6.43	1.36, 30.28	6.70	0.010	0.008	0.293	0.010	10.49
PTSD × Both Concussion Hx and Combat Exp	6.07	2.15, 17.18	12.63	0.000	<0.001	0.402	0.000	2.08
PTSD × Concussion Hx	2.13	0.762, 5.94	2.12	0.146	0.113	0.165	0.146	716.07 ^#^
PTSD × Multiple Concussion Hx	1.53	0.577, 4.04	0.73	0.392	0.271	0.097	0.392	8.06 ^#^
PTSD as a predictor in relation to the outcome variables								
PTSD × Either Depression ^j^ or Suicide Ideation ^k^	8.16	1.74, 38.25	8.95	0.003	0.002	0.339	0.003	5.37
PTSD × Either Anxiety ^l^ or Suicide Ideation	8.16	1.74, 38.25	8.95	0.003	0.002	0.339	0.003	5.37
PTSD × Any 1 of the Big 3 ^m^	6.43	1.36, 30.28	6.70	0.010	0.008	0.293	0.010	10.49
PTSD × Both Anxiety and Suicide Ideation	6.09	2.06, 18.07	11.76	0.000	0.000	0.388	0.000	2.26
PTSD × Suicide Ideation	5.64	1.84, 17.31	10.13	0.001	0.001	0.360	0.001	2.59
PTSD × All 3 of the Big 3	5.19	1.75, 15.39	9.65	0.002	0.003	0.352	0.002	2.60
PTSD × Any 2 of the Big 3	4.83	1.66, 14.07	8.99	0.003	0.003	0.340	0.003	2.68
PTSD × Both Depression and Anxiety	4.18	1.53, 11.43	8.20	0.004	0.005	0.324	0.004	2.70
PTSD × Both Depression and Suicide Ideation	3.80	1.40, 10.32	7.21	0.007	0.008	0.304	0.007	3.08
PTSD × Anxiety	3.77	1.39, 10.23	7.09	0.008	0.008	0.302	0.008	3.12
PTSD × Depression	3.11	1.11, 8.68	4.88	0.027	0.024	0.250	0.027	9.55
PTSD × Either Depression or Anxiety	3.05	1.05, 8.84	4.41	0.036	0.030	0.238	0.036	32.46

^a^ Odds ratio; ^b^ Confidence interval; ^c^ Chi-squared, ^d^ Cramer’s V test; ^e^ Skepticism limit (based on the Fisher’s Exact Test (one-sided) *p*-value); ^f^ Post-traumatic stress disorder; ^g^ Combat experience; ^h^ History; ^i^ Concussion history, ^j^ Positive for depression as per a 9-item Patient Health Questionnaire (depression) ≥10; ^k^ Positive for suicide ideation as per the Columbia-Suicide Severity Rating Scale—“Yes” response to item 2 or item 6 of the survey; ^l^ Positive for anxiety as per a Generalized Anxiety Disorder (7 Items)score of ≥10; ^m^ Big 3—combination of depression, anxiety, and suicide ideation; ^#^ Due to a *p*-value > 0.05, an advocacy limit was calculated.

**Table 7:** Results of 2 × 2 cross-tabulation analyses for variable combinations for PTSD-positive veterans.

Variable	PTSD ^a^ Condition	OR ^b^	95% CI ^c^ for OR	χ^2 d^	*p*-Value for χ^2^	Fisher’s Exact Test (1-Sided) *p*-Value	V ^e^	*p*-Value for V	AL ^f^ (AL, 1)
Any 1 of the Big 3 ^g^ × Multiple Concussion Hx	Positive	4.60 *	0.199, 106.30	1.71	0.191	0.303	0.261	0.191	2951.60
	Negative	1.18	0.357, 3.91	0.750	0.784	0.515	0.038	0.784	1.97
Either Anxiety ^h^ or Suicide Ideation ^i^ × Multiple Concussion Hx ^j^	Positive	4.60 *	0.199, 106.30	1.71	0.191	0.303	0.261	0.191	2951.60
	Negative	0.833	0.264, 2.63	0.970	0.756	0.491	0.043	0.756	0.473 ^#^
Any 1 of the Big 3 × Both Multiple Concussion Hx and Combat Exp ^k^	Positive	3.28 *	0.141, 76.04	1.22	0.269	0.400	0.221	0.269	252.45
	Negative	5.75 *	0.293, 112.89	2.42	0.120	0.158	0.214	0.120	43,860.45
Either Anxiety or Suicide Ideation × Both Multiple Concussion Hx and Combat Exp	Positive	3.28 *	0.141, 76.04	1.22	0.269	0.400	0.221	0.269	252.45
	Negative	2.25	0.218, 23.19	0.486	0.486	0.445	0.096	0.486	39.85
Any 1 of the Big 3 × Concussion Hx ^l^	Positive	2.83	0.152, 52.74	0.522	0.470	0.490	0.144	0.470	117.52
	Negative	2.22	0.708, 6.97	1.90	0.168	0.138	0.189	0.168	505.78
Either Anxiety or Suicide Ideation × Concussion Hx	Positive	2.83	0.152, 52.74	0.522	0.470	0.490	0.144	0.470	117.52
	Negative	1.90	0.628, 5.75	1.30	0.254	0.195	0.157	0.254	47.88
Any 1 of the Big 3 × Both Concussion Hx and Combat Exp	Positive	1.88	0.130, 34.13	0.185	0.667	0.600	0.086	0.667	24.77
	Negative	8.61	1.02, 73.02	5.02	0.024	0.022	0.310	0.024	6,226,740 ^†^
Either Anxiety or Suicide Ideation × Both Concussion Hx and Combat Exp	Positive	1.88	0.103, 34.13	0.185	0.667	0.600	0.086	0.667	13.99
	Negative	4.76	0.926, 24.48	3.94	0.047	0.046	0.273	0.047	N/A
Any 1 of the Big 3 × Either Concussion Hx or Combat Exp	Positive	1.72 *	0.063, 46.95	0.189	0.664	0.843	0.087	0.664	9.29
	Negative	2.16	0.674, 6.92	1.71	0.191	0.157	0.180	0.191	237.95
Any 1 of the Big 3 × Either Multiple Concussion Hx or Combat Exp	Positive	1.72 *	0.063, 46.95	0.189	0.664	0.843	0.087	0.664	9.29
	Negative	2.04	0.650, 6.39	1.51	0.219	0.174	0.169	0.219	105.08
Either Anxiety or Suicide Ideation × Either Concussion Hx or Combat Exp	Positive	1.72 *	0.063, 46.95	0.189	0.664	0.843	0.087	0.664	9.29
	Negative	2.04	0.650, 6.39	1.51	0.219	0.174	0.169	0.219	105.08
Either Anxiety or Suicide Ideation × Either Multiple Concussion Hx or Combat Exp	Positive	1.72 *	0.063, 46.95	0.189	0.664	0.843	0.087	0.664	9.29
	Negative	1.82	0.597, 5.54	1.12	0.291	0.219	0.145	0.291	28.83
Both Depression ^m^ and Suicide Ideation × Combat Exp	Positive	1.63	0.190, 13.93	0.198	0.656	0.532	0.089	0.656	7.78
	Negative	1.08	0.303, 3.87	0.015	0.902	0.574	0.017	0.902	1.38
Both Depression and Suicide Ideation × Either Concussion Hx or Combat Exp	Positive	1.56	0.086, 28.15	0.091	0.763	0.650	0.060	0.763	6.11
	Negative	1.79	0.481, 6.69	0.767	0.381	0.292	0.120	0.381	18.38
Both Depression and Suicide Ideation × Either Multiple Concussion Hx or Combat Exp	Positive	1.56	0.086, 28.15	0.091	0.763	0.650	0.060	0.763	6.11
	Negative	1.62	0.464, 5.65	0.576	0.448	0.329	0.104	0.448	9.62

^a^ Post-traumatic stress disorder; ^b^ Odds ratio; ^c^ confidence interval; ^d^ Chi-squared; ^e^ Cramer’s V test; ^f^ Advocacy limit (1, AL); ^g^ Big 3—combination of depression, anxiety, and suicide ideation; ^h^ Positive for anxiety as per a Generalized Anxiety Disorder (7 Items) score of ≥10; ^i^ Positive for suicide ideation as per the Columbia-Suicide Severity Rating Scale—“Yes” response to item 2 or item 6 of the survey; ^j^ History; ^k^ Combat experience; ^l^ Concussion history; ^m^ Positive for depression as per a 9-item Patient Health Questionnaire (depression) ≥10. * One cell in the 2 × 2 cross-tabulation table has zero cases; therefore, 0.5 was added to each of the cells so arithmetic could be computed to achieve extrapolated results. ^#^ Because the OR < 1.0, this result needs the output to be inverted (AI, 1). ^†^ Due to the Fisher’s Exact Test (one-sided) being <0.05, the skepticism limit was calculated (N/A)—with the lower limit of the OR 95% CI < 1, it was not possible to calculate the SL for these variables.

## Data Availability

The raw data supporting the conclusions of this article will be made available by the authors upon request.

## Abbreviations

The following abbreviations are used in this manuscript:

PTSD	Post-traumatic stress disorder
GWI	Global Well-being Index
PHQ-9	9-Item Patient Health Questionnaire
GAD-7	Generalized Anxiety Disorder (7 Items)
C-SSRS	Columbia-Suicide Severity Rating Scale
CPI	Critical prior interval
VA	Department of Veterans Affairs
mTBI	Mild traumatic brain injury
ACRM	American Congress of Rehabilitation Medicine
CISG	Concussion in Sport Group
ICC	Intraclass Correlation Coefficient
OR	Odds ratio
95% CI	95% confidence intervals
*χ*^2^	Chi-square
V	Cramer’s V test
AL	Advocacy limit
SL	Skepticism limit
AI	Artificial intelligence
Big 3	PHQ-9; GAD-7; C-SSRS
CTE	Chronic Traumatic Encephalopathy (CTE)
